# A scoping review and critical evaluation of the methodological quality of clinical practice guidelines on nutrition in the preconception

**DOI:** 10.3389/fnut.2023.1122289

**Published:** 2023-10-19

**Authors:** Mónica Ancira-Moreno, Soraya Burrola-Méndez, Cinthya Muñoz-Manrique, Isabel Omaña-Guzmán, Elizabeth Hoyos-Loya, Alejandra Trejo-Domínguez, Sonia Hernández-Cordero, Mónica Mazariegos, Natalia Smith, Loredana Tavano-Colaizzi, Jennifer Mier-Cabrera, Fermín Avendaño-Álvarez, Salvador Espino y Sosa, Karla Muciño-Sandoval, Lizeth Ibarra-González, Cristina Medina-Avilés

**Affiliations:** ^1^Department of Health, Universidad Iberoamericana, Mexico City, Mexico; ^2^Maternal and Child Health and Nutrition Network (MaCHiNNe), Observatorio Materno Infantil, Universidad Iberoamericana, Mexico City, Mexico; ^3^Coordination of Nutrition and Bioprogramming, Instituto Nacional de Perinatología, Mexico City, Mexico; ^4^Pediatric Obesity Clinic and Wellness Unit, Hospital General de México “Dr. Eduardo Liceaga”, Mexico City, Mexico; ^5^Research Center for Equitable Development EQUIDE, Universidad Iberoamericana, Mexico City, Mexico; ^6^Research Center for the Prevention of Chronic Diseases (CIIPEC), Institute of Nutrition of Central America and Panama (INCAP), Guatemala City, Guatemala; ^7^Department of Pediatrics, University of California, San Francisco, San Francisco, CA, United States; ^8^Clinical Research Branch, Instituto Nacional de Perinatología Isidro Espinosa de los Reyes, Mexico City, Mexico; ^9^Nutrition and Dietetics Service, Instituto Nacional de Perinatología, Mexico City, Mexico; ^10^Sub-Direction of Gynecology and Obstetrics Instituto Nacional de Perinatología, Mexico City, Mexico

**Keywords:** AGREE II, clinical practice guidelines, nutrition, preconception, methodological quality appraisal

## Abstract

**Introduction:**

Clinical practice guidelines (CPGs) contain recommendations for specific clinical circumstances, including maternal malnutrition. This study aimed to identify the CPGs that provide recommendations for preventing, diagnosing, and treating women’s malnutrition. Additionally, we sought to assess the methodological quality using the Appraisal of Guidelines for Research and Evaluation (AGREE II) instrument.

**Methods:**

An online search for CPGs was performed, looking for those that contained lifestyle and nutritional recommendations to prevent, diagnose and treat malnutrition in women during the preconception period using PubMed and different websites. The reviewers utilized the AGREE II instrument to appraise the quality of the CPGs. We defined high-quality guidelines with a final score of > 70%.

**Results:**

The titles and abstracts from 30 guidelines were screened for inclusion, of which 20 guidelines were fully reviewed for quality assessment. The overall quality assessment of CPGs was 73%, and only 55% reached a high-quality classification. The domains in the guidelines classified as high-quality had the highest scores in “Scope and Purpose” and “Clarity of Presentation” with a median of 98.5 and 93%, respectively.

**Discussion:**

Further assessment is needed to improve the quality of the guidelines, which is an opportunity to strengthen them, especially in the domains with the lowest scores.

## 1. Introduction

Maternal malnutrition is associated with irreversible negative health outcomes for the mother–child binomial in the medium and long term (1). Women’s health and nutrition status before pregnancy is crucial in determining gestational weight gain, pregnancy health, and birth outcomes (2). Nevertheless, the preconception nutritional status has been overlooked despite its importance; poor+ nutrition in the preconception period is women’s least studied stage of life (3).

Globally, more than one billion women experience at least one form of malnutrition. The prevalence of underweight in women of reproductive age in 2014 was 9.7%, and substantial burdens persist across Asia and Africa, reaching 24% in South Asia (4). In Southeast and South Asia, maternal short stature (< 150 cm) affects 40–70% of women. Latin America and the Caribbean, Pacific Islands, and the Middle East bear a significant burden of overweight and obesity, with even higher prevalence observed in regions like South Asia (5). In addition, one-third of women of reproductive age in lower-middle-income countries are anemic, and vitamin D deficiency is re-emerging as a significant global health issue (6, 7). Recent studies have linked the above-mentioned conditions with several clinical conditions in pregnancy (e.g., preeclampsia, gestational diabetes, higher incidence of cesarean section, preterm birth, etc.) (8).

Clinical practice guidelines (CPGs) provide recommendations that are designed to aid healthcare providers, physicians, and patients in making informed decisions regarding appropriate healthcare for specific clinical circumstances, such as the supplementation with folate, iron, and folic acid, and weight management of women with obesity in pregnancy (9); as well as recommendations for nutritional assessment, healthy diet, dietary modifications, nutritional supplementation, or any nutritional or lifestyle recommendations given in primary care and other health care areas. However, CPGs vary among countries or regions, and some of them do not meet the basic quality standards (10, 11). Furthermore, there is often a lack of regular updates to guidelines, which means that they may not always remain up-to-date and fail to incorporate the most current evidence (8).

The Appraisal of Guidelines for Research and Evaluation Instrument (AGREE II) was developed to address the issue of quality variability in CPGs. Its main objectives are to establish a framework for assessing guideline quality, offer a methodological approach for guideline development, and provide guidance on what information should be included and how it should be reported. The AGREE II instrument can be applied to any health or disease-related guidelines, including preconception, pregnancy, the postpartum period, and other stages of women’s lives (12).

High-quality CPGs benefit the reduction of issues related to poor nutrition in the preconception period. This study aimed to identify the CPGs that include recommendations for preventing, diagnosing, and treating women’s malnutrition and to evaluate the methodological quality of the included guidelines using the AGREE II instrument.

## 2. Materials and methods

### 2.1. Data sources and search strategy

We thoroughly assessed CPGs, including lifestyle and nutritional recommendations to prevent, diagnose and treat malnutrition in the preconception period. Our study incorporated CPGs, standard references, and position statements that provided recommendations on various aspects of nutritional assessment (including anthropometric measurements, biochemical data, clinical history, and lifestyle factors), healthy diet, dietary modifications, nutritional supplementation, and other nutritional or lifestyle recommendations.

The review process consisted of five stages. For our study, we utilized the framework initially proposed by Arksey and O’Malley (13), which was further refined by Levac et al. (14) and the Joanna Briggs Institute (15). We added one last step to assess the quality of the CPGs using the AGREE II instrument (12).

We performed two types of searches for our study. The first search involved a systematic search in a single bibliographic database ^1^ using the algorithm outlined in Table 1 and filters for guidelines and practice guidelines. The second search involved a manual search on guideline-related websites of national and international agencies and societies focused on child health and nutrition. We used key terms from the PubMed algorithm, individually and combined in English and Spanish, for this manual search.

**Table 1:** Search algorithm.

Algorithm	Limits
(“preconception period” OR preconception) AND (“Preconception Care”[MeSH^†^] OR “Nutrition Assessment”[MeSH] OR “Nutrition Therapy”[MeSH] OR “prevention and control” [Subheading] OR “Health Promotion”[MeSH]) AND (“Malnutrition”[MeSH] OR “Body Weight”[MeSH] OR “Anemia”[MeSH] OR “Deficiency Diseases”[MeSH] OR “Nutrition Disorders”[MeSH] OR “Nutritional Physiological Phenomena”[MeSH])	Article Type (Guideline, Practice Guideline); Languages (English, Spanish); Publication date (From 2008/1/1 to 2021/2/1)

† MeSH, Medical subject headings.

### 2.2. Studies selection

#### 2.2.1. Inclusion criteria

The included documents met the following eligibility criteria: (i) they were international and national CPGs, standard references, or position statements; (ii) they were written in English or Spanish; (iii) they were published between January 2008 and February 2021, considering the publication of The Lancet’s Maternal and Child Undernutrition Series.

#### 2.2.2. Exclusion criteria

The exclusion criteria encompassed opinions or editorials, articles published as communication tools, and clinical practice guidelines (CPGs) focused solely on lifestyle and nutrition recommendations related to a specific pathology or its associated complications. After importing the identified studies into Excel, any duplicate entries were removed.

### 2.3. Quality assessment

The evaluation process involved the participation of authors, including dietitians and physicians. Two of the authors (CMM, MAM) independently reviewed the titles and abstracts of each study to determine their eligibility for inclusion. In the event of disagreements, another author (SBM) evaluated the guideline to provide a final decision. We obtained full-text copies of the potentially eligible documents; one of them was independently assessed by two authors to determine if they met the inclusion criteria. In case of disagreements, a third author was assigned to determine the final inclusion of the study.

The AGREE II instrument assesses a CPG’s development in terms of its quality, rigor, and transparency. It comprises six domains (Table 2) consisting of 23 key items in total. Each item within the instrument is assessed using a seven-point Likert rating scale, ranging from one (Strongly Disagree) to seven (Strongly Agree), as defined in the AGREE II User’s Manual (10). The overall scores of each of the six domains were calculated by adding all their corresponding items and scaling the total as a proportion of the maximum possible score for that domain (max score = 100). An overall assessment score of > 70% indicated high quality in the guidelines (10). The quality of each CPGs was independently evaluated by two authors (SES, LTC, AT, FAA, MAM) using the online AGREE platform “My AGREE PLUS.”

**Table 2:** The Appraisal of Guidelines for Research and Evaluation Instrument II domains and content.

Domain	Content
1. Scope and purpose	Related to the overall aim of the guideline
2. Stakeholder involvement	Measures the extent to which the guideline was developed by the appropriate stakeholders
3. Rigour of development	Focuses on the methodology employed for evidence collection and synthesis the evidence
4. Clarity of presentation	Assesses the language, structure, and format of the guideline
5. Applicability	Examines the practical implications of implementing the guideline
6. Editorial independence	Evaluates if the formulation of the recommendations is unbiased by competing interests

Extracted from the AGREE II instrument (16).

### 2.4. Data analysis

The means and median scores for each domain of the AGREE II instrument were computed to determine the most critical domains across the different guidelines. The overall quality of each guideline was assessed by applying a threshold of 70% for the final score of each domain. Data collection and extraction were performed using Microsoft Excel 2021, version 16.57. This study did not require ethical approval or consent.

## 3. Results

**Figure 1:**
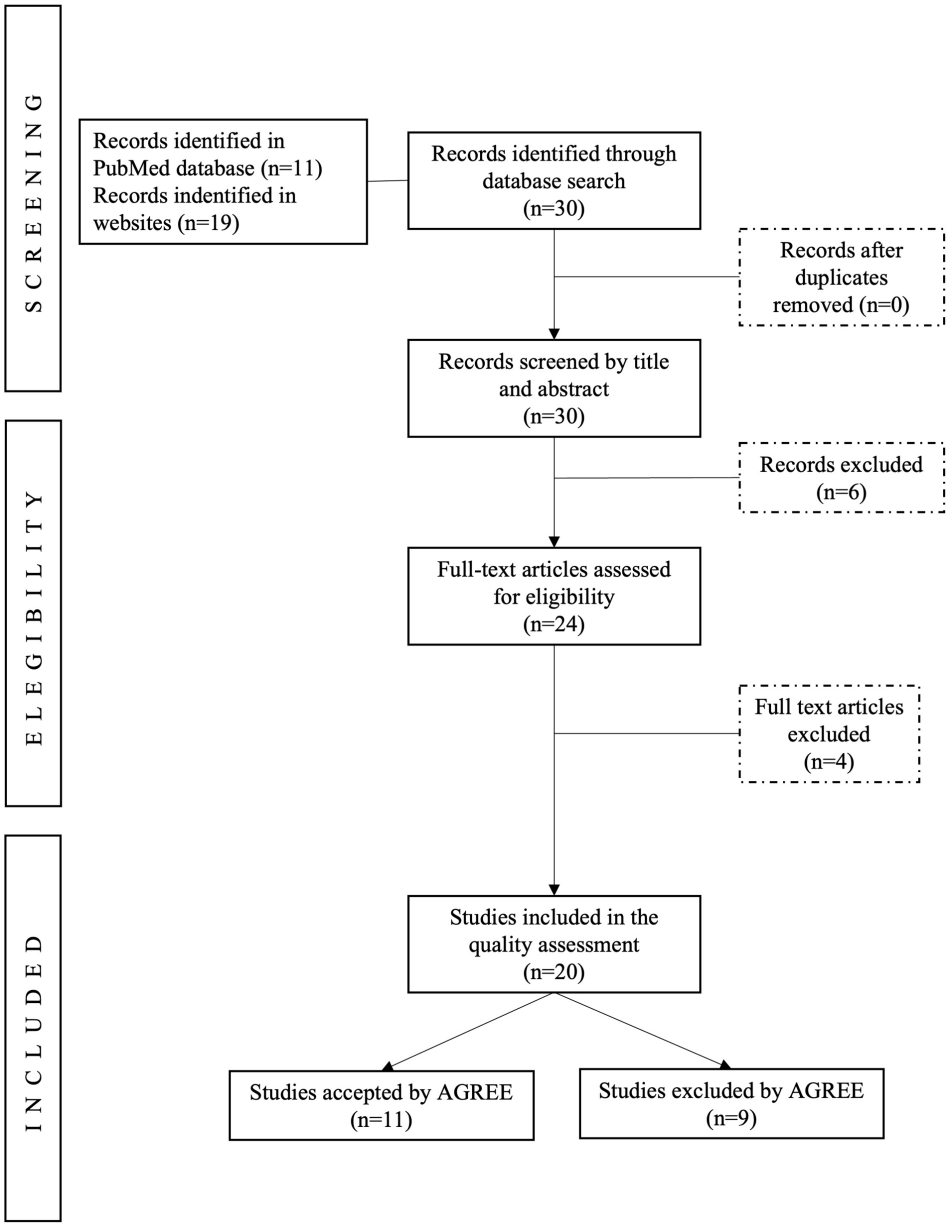
PRISMA flow diagram of literature sources and review process.

A summary of the results is shown in Figure 1, which was yielded by the keyword combinations with PubMed and other websites. We started the eligibility process after collecting all the results and omitting duplicated articles. The titles and abstracts from 30 guidelines were screened for inclusion, of which 20 guidelines were fully reviewed for quality assessment.

Of the 20 PCGs, six were related to prenatal care for pregnancy and six to weight control, overweight and obesity in women of reproductive age and during pregnancy, five of the guidelines were focused on supplementation of iron, folic acid, calcium or vitamin K, and the rest of the guidelines provided recommendations for healthy eating and lifestyle and for preconception management in women with diabetes.

Supplementary material 1 shows the general characteristics of the included guidelines, such as reference clinical guidelines, supporting organization, year, region, number of references and target audience. The main supporting organization is the World Health Organization (WHO) (17–21) NICE (22–24), the Royal College of Obstetricians and Gynecologists (25, 26) and other Societies, Colleges and Departments of Health.

Studies were published from 2009 to 2021. Of the 20 guidelines, six were internationally developed (17–21, 27), and the others were created in six different countries, including the United Kingdom (22–26), Canada (28–30), the United States of America (USA) (31, 32), Australia (33, 34), Latvia (35) and Poland (36) with one each.

The mean of references was 89.3 (Min:13 Max:239); however, three guides by NICE did not specify their references (22–24). The guidelines were designed for different target audiences, and the main ones were healthcare providers. Some guides directed their guidelines towards policymakers, expert advisers, government officials, scientists, the food industry and organizations of nutrition actions for public health.

### 3.1. Quality of guidelines according to the AGREE II domains.

Table 3 presents the scores for each domain and the final quality evaluation of all CPGs. The overall quality assessment was 73% (range = 39–100), and the median was 83% (range = 17–100). 75% (*n* = 15) reached a high-quality classification. About the domains, three of them had a score of > 70%. The domain with the highest score was “Clarity of presentation,” with a mean of 88.5% (range = 50–100), and “Scope and purpose,” with a mean of 87% (range = 39–100), while the lowest was “Applicability” with a mean of 69.9% (range = 4–100). High-quality guidelines had a higher evaluation in “Scope and Purpose” and “Clarity of Presentation” with a mean of 97.3% (range = 39–100) and 94.9% (range = 50–100), respectively; meanwhile, the domain with the lowest score was “Applicability” with a mean of 81.5% (range = 60–100). In the guidelines classified as low quality, the domain with the lowest score was “Applicability” with a mean of 35% (score = 4–100) and “Rigour of development” with 36.8% (score = 21–100).

**Figure 2:**
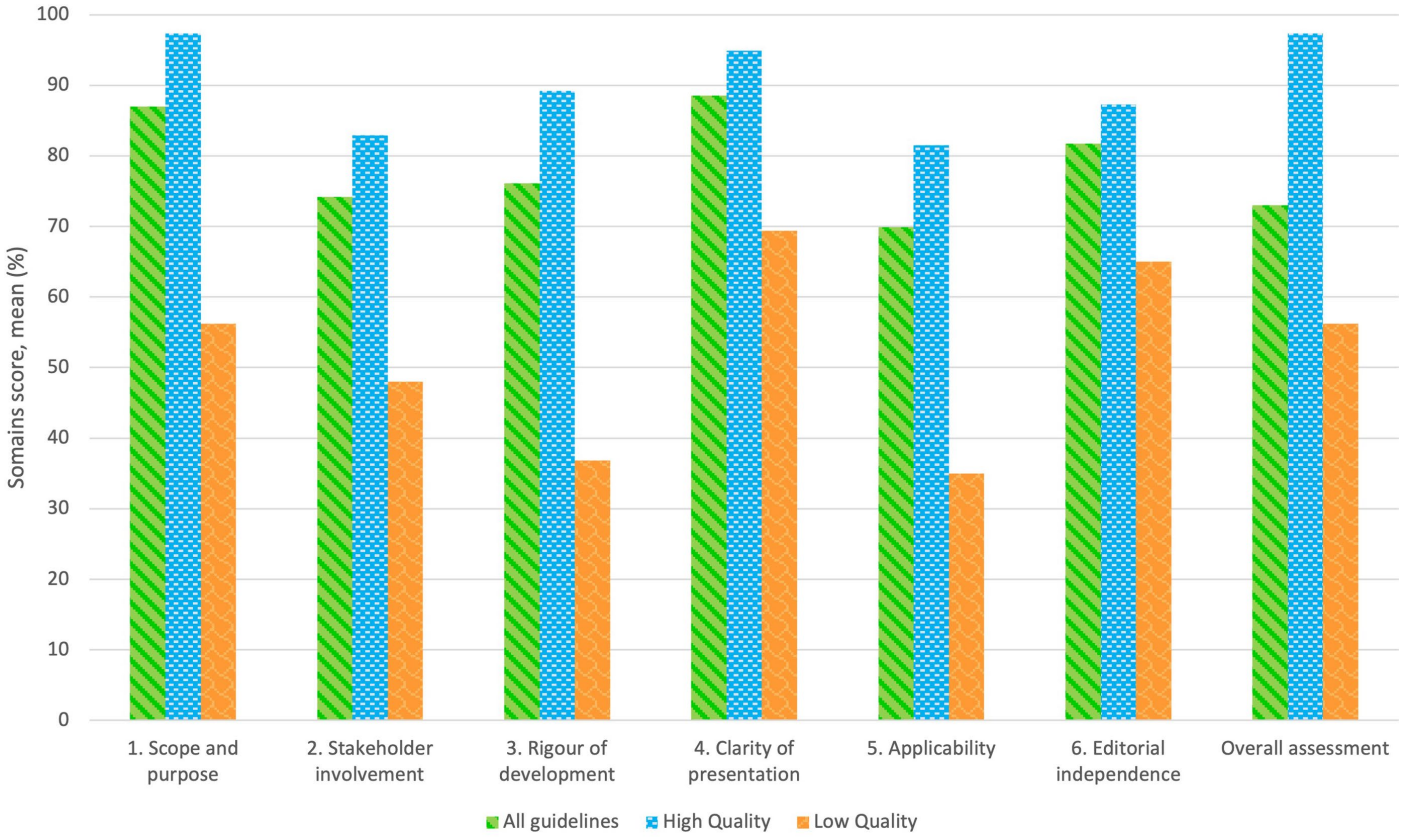
Average quality score by each domain of AGREE II for all included guidelines.

Two clinical guidelines developed by NICE, “Antenatal care for uncomplicated pregnancies” in 2019 (22) and “Weight management before, during, and after pregnancy” in 2010 (24), had the highest score (more than 90% in all the evaluated domains); while the clinical guidelines by Bomba-Opoń D. et al. (36), the Royal Australian and New Zealand College of Obstetricians and Gynecologists (34), the American College of Obstetricians and Gynecologists and the American Society for Reproductive Medicine (32), McAuliffe FM et al. (27) and Australian Government Department of Health (33) had an overall low quality with 39, 50, 56, 67 and 69%, respectively. Therefore, they are not recommended according to the AGREE II assessment tool. The average quality scores of each domain of the AGREE II instrument by all guidelines, high-quality guidelines, and low-quality guidelines are shown in Figure 2.

#### 3.1.1. Scope and purpose domain

For the “Scope and Purpose” domain, 75% (*n* = 15) of the guidelines received a score > 80%. The lowest scores (below ≤ 50%) were achieved by “Folate supplementation during the preconception period, pregnancy and puerperium” (2017) (36) with 39%, “Pre-pregnancy counseling” by The Royal Australian and New Zealand College of Obstetricians and Gynecologists (2021) (34) and The American College of Obstetricians and Gynecologists and the American Society for Reproductive Medicine (2019) (32) with 50 and 56%, respectively.

**Table 3:** Appraisal of Guidelines for Research and Evaluation (AGREE) II version result for clinical practice guidelines.

Clinical guideline	AGREE II domains (%)							
	Scope and purpose	Stakeholder involvement	Rigour of development	Clarity of presentation	Applicability	Editorial independence	Overall assessment	Quality of guidelines
Antenatal care for uncomplicated pregnancies (22)	100	97	100	100	94	92	100	High quality
Canadian Adult Obesity Clinical Practice Guidelines: Weight Management Over the Reproductive Years for Adult Women Living with Obesity (30)	94	86	75	92	88	58	94	High quality
Care of Women with Obesity in Pregnancy: Green-top Guideline No. 72 (26)	86	58	97	97	65	96	86	High quality
Clinical Practice Guidelines: pregnancy care (33)	69	56	56	83	48	71	69	Low quality
Diabetes in pregnancy: management from preconception to the postnatal period (23)	100	100	84	100	98	92	100	High quality
Folate supplementation during the preconception period, pregnancy and puerperium. Polish Society of Gynecologists and Obstetricians Guidelines (36)	39	28	21	64	4	8	39	Low quality
Guideline No. 391-Pregnancy and Maternal Obesity Part 1: Pre-conception and Prenatal Care (29)	97	83	100	94	60	83	97	High quality
Guideline: intermittent iron and folic acid supplementation in menstruating women (17)	100	83	96	92	100	83	100	High quality
Guideline: optimal serum and red blood cell folate concentrations in women of reproductive age for prevention of neural tube defects (18)	100	81	99	97	100	92	100	High quality
Guideline: sodium intake for adults and children (19)	100	83	98	100	88	100	100	High quality
Guideline: sugars intake for adults and children (19, 20)	100	83	98	100	100	100	100	High quality
Management of Women with Obesity in Pregnancy (25)	100	89	92	94	83	92	100	High quality
Obesity and reproduction (28)	94	56	75	83	40	50	94	High quality
Practice parameter update: management issues for women with epilepsy--focus on pregnancy (an evidence-based review): vitamin K, folic acid, blood levels, and breastfeeding (31)	100	81	73	92	75	75	100	High quality
Pre-pregnancy counseling (34)	50	50	49	50	38	50	50	Low quality
Pre-pregnancy counseling (32)	56	53	34	86	52	100	56	Low quality
Prevention of noncommunicable diseases by interventions in the preconception period: A FIGO position paper for action by healthcare practitioners (27)	67	53	24	64	33	96	67	Low quality
Proper maternal nutrition during pregnancy planning and pregnancy: a healthy start in life (35)	89	81	63	83	63	96	89	High quality
Weight management before, during, and after pregnancy (24)	100	100	92	100	94	100	100	High quality
WHO recommendation on calcium supplementation before pregnancy for the prevention of pre-eclampsia and its complications (21)	100	83	96	100	75	100	100	High quality
**Mean (range)**	**87.0 (range 39 – 100%)**	**74.2 (range 28 – 100%)**	**76.1 (range 21–100%)**	**88.5 (range 50 – 100%)**	**69.9 (range 4 – 100%)**	**81.7 (range 8 – 100%)**	**73.0 (range 17 – 100%)**	
**Median (range)**	**98.5 (range 39 – 100%)**	**82 (range 28 – 100%)**	**88 (range 21 – 100%)**	**93 (range 50 – 100%)**	**75 (range 4 – 100%)**	**92 (range 8 – 100%)**	**83 (range 17 – 100%)**	

#### 3.1.2. Stakeholder involvement

Domain 2, the mean score was 74.2% (range = 28–100) and the median score of 82% (range = 28–100%). Of the guidelines, 10% (*n* = 12) had a maximum score of 100% in “Diabetes in pregnancy: management from preconception to the postnatal period” (2020) (23) and “Weight management before, during, and after pregnancy” (2010) (24). The “Folate supplementation during the preconception period, pregnancy and puerperium” (2017) (36) was the only guideline that scored below 50%.

#### 3.1.3. Rigour of development

For the 20 sets of guidelines, the mean AGREE II score for the domain “Rigour and development” was 76.1% (range = 21–100). The highest score for this domain was observed in two CPGs (10%): “Antenatal care for uncomplicated pregnancies” (22) and “Guideline No. 391-Pregnancy and Maternal Obesity Part 1: Pre-conception and Prenatal Care” (29), both in 2019. Of the guidelines, 70% received a score higher than 70, and 15% (*n* = 3) scored below 50% (27, 32, 34). “Prevention of noncommunicable diseases by interventions in the preconception period: A FIGO position paper for action by healthcare practitioners” (2020) (27) had the lowest score in this domain.

#### 3.1.4. Clarity of presentation

Compared with the others, this domain obtained the highest score with a mean of 88.5% (range = 50–99) and median score of 93% (range = 50–100). The scores established for this domain were high for all the guidelines; 85% (*n* = 17) of them scored > 70%.

#### 3.1.5. Applicability

This domain obtained the lowest score with a mean of 69.9% (range = 4–100%) and a median score of 75% (range = 4–100). Half of the guidelines (50%) analyzed received a score > 70%. The nine reached an evaluation > 70% and “Obesity and Reproduction” (2018) (28), “Pre-pregnancy counseling” (2019) (34), “Prevention of noncommunicable diseases by interventions in the preconception period: A FIGO position paper for action by healthcare practitioners” (2020) (27) and “Folate supplementation during the preconception period, pregnancy and puerperium. Polish Society of Gynecologists and Obstetricians Guidelines” (2017) (36) had a score lower score with 40, 38, 33, 4%, respectively.

#### 3.1.6. Editorial independence

On the “Editorial independence” domain, the guidelines obtained a mean AGREE II score of 81.7% (range = 8–100). Fourteen (70%) received a score higher than 80%. Bomba-Opoń D et al. (36) ‘s guideline was the only one that scored equal to 8%.

## 4. Discussion

Most of the CPGs we found included recommendations for managing obesity and the prescription of supplements. Nevertheless, few guidelines have been developed to make recommendations about iron and folic acid supplementation, even though anemia is one of the most common forms of malnutrition in this group of women (6, 7). In addition, elaborated guides for optimizing weight were not identified despite the important role that nutritional status during preconception plays in determining health outcomes in pregnant women (2).

Our main findings revealed that only 55% of the CPGs were evaluated as high quality, while the domain scores were between high- and low-quality CPGs. High-quality CPGs had a higher evaluation in the classifications of “Scope and Purpose” (median = 98.5%, range = 39–100) and “Clarity of Presentation” (median = 93%, range = 50–100). Low-quality CPGs had a higher score in the classification of “Clarity of presentation” (median = 93%, range 50–100) and “Editorial Independence” (median = 92%, range 8–100). In the guidelines classified as high quality and low quality, the domain with the lowest score was “Applicability,” with a median of 48% (range = 60–100) and 75% (score = 4–100%), respectively. Our results agree with other quality assessments of CPGs using the AGREE II instrument (37).

According to the AGREE II instrument, several quality domains need to be improved and prioritized; in this context, domains 5 and 2, which are “Applicability” and “Stakeholder involvement,” obtained the lowest mean (69.9 and 74.2%, respectively) in most of the guidelines. The “Applicability” domain has been reported to be related to implementing the guidelines by health professionals in daily clinical practice (12). This situation may be a key to understanding the gap between knowledge and implementation of CPGs, in addition to the potential implications on the clinical practice and the nutritional status of women. In our context, it is necessary the development of robust, comprehensive, and high-quality guidelines for a healthy lifestyle in the preconception period (38).

This study has different limitations. First, our systemic search was exclusively conducted in one database (PubMed) which may have limited the search for developing countries. Secondly, the search was restricted to CPGs published in Spanish or English. It is important to acknowledge certain limitations when interpreting these results because the geographical generalizability may be limited considering the under-representation from low and middle-income regions such as Asia, Africa, Latin America and Caribe.

Only a few methodologies have been designed to assess the quality of CPGs. AGREE II provides elements that allow for developing and implementing initiatives to improve healthcare quality. We recommend this instrument that guideline developers, clinicians, researchers, and policymakers consider and utilize the AGREE II tool, as it is a comprehensive and user-friendly instrument that can be adapted to specific populations, injuries, or diseases (39).

There is a gap in the evidence of the different forms of malnutrition in the preconception period, and sometimes, the guidelines have yet to be adapted to new contexts, like the pandemic caused by Coronavirus SARS-CoV-2 in 2020 (8). To our knowledge, this is the first study that evaluates the quality of CPGs for the preconception period and the importance of including different health professionals, such as dietitians, related to the preconception in this evaluation process.

## 5. Conclusion

AGREE II tool provides a framework to develop guidelines and an instrument to review their quality. Further assessment is needed to improve the quality guidelines, which is an opportunity to strengthen them, especially in the domains where the scores were the lowest. We recommend using the AGREE II instrument by all health professionals since it can be applied easily and in detail. This instrument also allows an analytical evaluation before implementing the given guidelines, which would support the making of decisions around the health system of a country or region. We need increased rigor in formulating guidelines to prevent, diagnose and treat malnutrition in all its forms during preconception, a critical period of life.

## Data availability statement

The raw data supporting the conclusions of this article will be made available by the authors, without undue reservation.

## Author contributions

SB-M, MA-M, NS, and CM-M conceptualized the study. NS, SB-M, MA-M, and IO-G conducted the methodology. SH-C, AT-D, and IO-G conducted the formal analysis. CM-M, NS, LT-C, FA-A, SE, KM-S, LI-G, and CM-A appraisers with experience in quality assessment of guidelines, scored each guideline using the AGREE II instrument. MA-M, NS, and SB-M conducted data curation. SB-M, MA-M, IO-G, and EH-L wrote the first draft on the manuscript. SB-M, MA-M, NS, IO-G, EH-L, and SH-C critically revised the manuscript. MA-M, SB-M, NS, IO-G, and SH-C supervised the project. MA-M and IO-G administered the project. MA-M funding acquisition. All authors have read and approved the final manuscript.

## Footnotes

^1^PubMed: https://ncbi.nlm.nih.gov/pubmed
