# Supplementary material for: A scoping review and critical evaluation of the methodological quality of clinical practice guidelines on nutrition in the preconception
Source: Front Nutr. 2023 Oct 19;10:1122289. doi: 10.3389/fnut.2023.1122289 (PMC10621738; doi:10.3389/fnut.2023.1122289)
Supplement: Supplementary file 1 [file Table_1.docx]

2018Supplementary Material

Evaluation of the methodological quality of clinical practice guidelines to prevent, diagnose, and treat malnutrition in the preconception period

**Mónica Ancira-Moreno*, Soraya Burrola-Méndez, Cinthya Muñoz-Manrique, Isabel Omaña-Guzman, Elizabeth Hoyos-Loya, Alejandra Trejo-Domínguez, Sonia Hernández-Cordero, Mónica Mazariegos, Natalia Smith, Loredana Tavano-Colaizzia, Jennifer Mier-Cabrera, Fermín Avendaño-Álvarez, Salvador Espino y Sosa, Karla Muciño-Sandoval, Lizeth Ibarra-González, Cristina Medina-Álvarez**

*** Correspondence:** Monica Ancira-Moreno: monica.ancira@ibero.mx

# Supplementary Data

General Characteristics of the 20 CPGs included in the analysis.

| **Reference** | **Clinical Guideline** | **Supporting organization** | **Year of publication** | **Region** | **Number of references** | **Target audience** |
| --- | --- | --- | --- | --- | --- | --- |
| Australian Government Department of Health (1) | Clinical Practice Guidelines: Pregnancy care | Australian Government Department of Health | 2019 | Australia | 67 | Health professionals |
| Bomba-Opoń D et al. (2) | Folate supplementation during the preconception period, pregnancy and puerperium. Polish Society of Gynecologists and Obstetricians Guidelines | Polish Society of Gynecologists and Obstetricians | 2017 | Poland | 30 | Not specified |
| CMACE/RCOG (3) | Management of Women with Obesity in Pregnancy | Royal College of Obstetricians and Gynecologists | 2010 | UK | 77 | Health professionals |
| Denison FC et al. (4) | Care of Women with Obesity in Pregnancy: Green-top Guideline No. 72 | Royal College of Obstetricians and Gynecologists | 2018 | UK | 202 | Clinicians |
| Harden CL et al. (5) | Practice parameter update: management issues for women with epilepsy--focus on pregnancy (an evidence-based review): vitamin K, folic acid, blood levels, and breastfeeding | American Academy of Neurology and American Epilepsy Society | 2009 | USA | 40 | Not specified |
| Mahutte N et al. (6) | Obesity and Reproduction | The Canadian Fertility & Andrology Society (CFAS) | 2018 | Canada | 239 | Health care providers |
| Maxwell C et al. (7) | Guideline No. 391-Pregnancy and Maternal Obesity Part 1: Pre-conception and Prenatal Care | The Society of Obstetricians and Gynecologists of Canada | 2019 | Canada | 178 | Health care providers |
| McAuliffe FM et al. (8) | Prevention of noncommunicable diseases by interventions in the preconception period: A FIGO position paper for action by healthcare practitioners | International Federation of Gynecology and Obstetrics (FIGO) | 2020 | International | 165 | Healthcare providers working with women with obesity |
| NICE (9) | Antenatal care for uncomplicated pregnancies | National Institute for Health and Care Excellence: Guidelines | 2019 | UK | Not specified | Clinicians |
| NICE (10) | Diabetes in pregnancy: management from preconception to the postnatal period | National Institute for Health and Care Excellence: Guidelines | 2020 | UK | Not specified | Health care providers |
| NICE (11) | Weight management before, during and after pregnancy | National Institute for Health and Care Excellence: Guidelines | 2010 | UK | Not specified | Health professionals working in weight management, fertility, pre-conception advice and care services, gynecology, and contraceptive services |
| Piccinini-VH et al. (12) | Canadian Adult Obesity Clinical Practice Guidelines: Weight Management Over the Reproductive Years for Adult Women Living with Obesity | Canadian Association of Bariatric Physicians and Surgeons | 2020 | Canada | 126 | Primary care providers |
| The American College of Obstetricians and Gynecologists and the American Society for Reproductive Medicine (13) | Pre-pregnancy counseling | The American College of Obstetricians and Gynecologists (ACOG)  American Society for Reproductive Medicine | 2019 | USA | 75 | Health care providers |
| The Royal Australian and New Zealand College of Obstetricians and Gynecologists (14) | Pre-pregnancy counseling | The Royal Australian and New Zealand College of Obstetricians and Gynecologists | 2021 | Australia | 13 | Health professionals |
| Meija L. et al (15) | Proper maternal nutrition during pregnancy planning and pregnancy: a healthy start in life | WHO Regional Office for Europe  Ministry of Health of Latvia | 2017 | Latvia | 27 | Not specified |
| WHO (16) | Guideline: intermittent iron and folic acid supplementation in menstruating women | WHO Guidelines Approved by the Guidelines Review Committee | 2011 | International | 32 | Policy-makers, expert advisers, and organizations of nutrition actions for public health |
| WHO (17) | Guideline: optimal serum and red blood cell folate concentrations in women of reproductive age for prevention of neural tube defects | WHO Guidelines Approved by the Guidelines Review Committee. | 2015 | International | 72 | Policy-makers, expert advisers and organizations of nutrition actions for public health |
| WHO (18) | Guideline: sodium intake for adults and children | WHO Guidelines Approved by the Guidelines Review Committee | 2012 | International | 62 | Policy-makers, expert advisers, and organizations of nutrition actions for public health |
| WHO (19) | Guideline: sugars intake for adults and children | WHO Guidelines Approved by the Guidelines Review Committee | 2015 | International | 61 | Government officials, scientists, food industry, and partners involved in public health nutrition |
| WHO (20) | WHO recommendation on calcium supplementation before pregnancy for the prevention of pre-eclampsia and its complications | WHO Guidelines Approved by the Guidelines Review Committee | 2020 | International | 35 | Health professionals responsible for developing national and local health protocols and directly providing care to pregnant women |

FIGO: International Federation of Gynecology and Obstetrics, NICE: National Institute of Health and Care Excellence, WHO: World Health Organization
